# Nanoarchitectonics for Hierarchical Fullerene Nanomaterials

**DOI:** 10.3390/nano11082146

**Published:** 2021-08-23

**Authors:** Subrata Maji, Lok Kumar Shrestha, Katsuhiko Ariga

**Affiliations:** 1 Center for Functional Sensor & Actuator (CFSN), Research Center for Functional Materials, National Institute for Materials Science (NIMS), 1-1 Namiki, Tsukuba 305-0044, Japan; MAJI.Subrata@nims.go.jp; 2 International Center for Materials Nanoarchitectonics (WPI-MANA), National Institute for Materials Science (NIMS), 1-1 Namiki, Tsukuba 305-0044, Japan; SHRESTHA.Lokkumar@nims.go.jp; 3 Graduate School of Frontier Sciences, The University of Tokyo, Kashiwa, Chiba 277-0827, Japan

**Keywords:** assembly, fullerene, hierarchical structure, interface, nanoarchitectonics, nanomaterial

## Abstract

Nanoarchitectonics is a universal concept to fabricate functional materials from nanoscale building units. Based on this concept, fabrications of functional materials with hierarchical structural motifs from simple nano units of fullerenes (C_60_ and C_70_ molecules) are described in this review article. Because fullerenes can be regarded as simple and fundamental building blocks with mono-elemental and zero-dimensional natures, these demonstrations for hierarchical functional structures impress the high capability of the nanoarchitectonics approaches. In fact, various hierarchical structures such as cubes with nanorods, hole-in-cube assemblies, face-selectively etched assemblies, and microstructures with mesoporous frameworks are fabricated by easy fabrication protocols. The fabricated fullerene assemblies have been used for various applications including volatile organic compound sensing, microparticle catching, supercapacitors, and photoluminescence systems.

## 1. Introduction

Materials sciences have been supported by various synthetic approaches including organic chemistry [1,2,3], inorganic chemistry [4,5,6], coordination chemistry [7,8,9], polymer chemistry [10,11,12], and others [13,14,15]. The prepared materials are utilized in many applications for the purpose of solving energy [16,17,18], environmental [19,20,21], and biomedical problems [22,23,24] upon social demands. Undoubtedly, the preparation and fabrication of high-performance materials are crucial issues. Based on analyses and characterizations with high-resolution techniques upon advanced nanotechnology [25,26,27], the importance of the regulation of nanoscale structures for better functions has been revealed [28,29,30]. Accordingly, nanoscale materials sciences have been paid much attention. Nanoscale materials in various dimensions such as quantum dots [31,32,33], nanoparticles [34,35,36], nanocrystals [37,38,39], nanotubes [40,41,42], nanorods/nanowires [42,43,44,45], nanosheets [46,47,48], graphene [49,50,51], and other two-dimensional materials [52,53,54] have been extensively investigated. Similarly, materials with internal nanostructures including mesoporous materials [55,56,57], zeolites [58,59,60], metal–organic frameworks [61,62,63], other coordination polymers [64,65,66], and covalent organic frameworks [67,68,69] have been actively explored.

**Figure 1:**
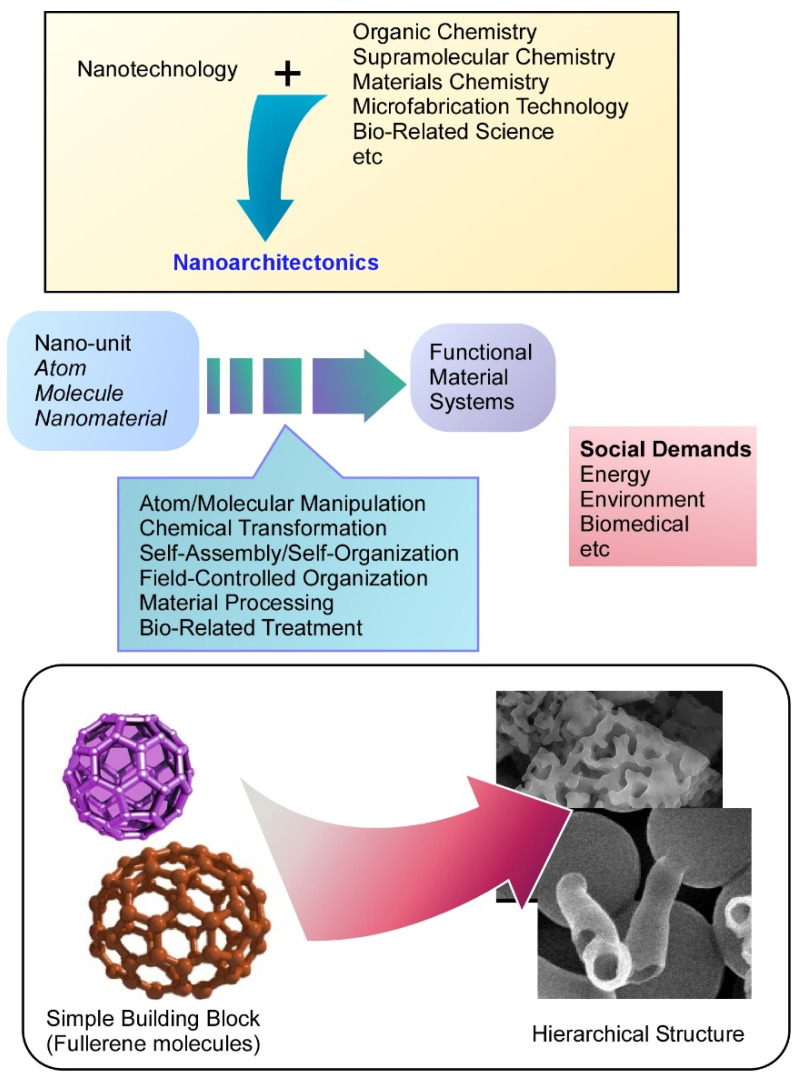
Nanoarchitectonics is a conceptual methodology to combine nanotechnology with other research fields, in which functional material systems are prepared from nanoscale units.

In addition to these nanomaterials and materials with internal nanostructures, materials prepared through assembly and aggregation of small units have also become important in the exploration of functional materials. Such materials have been prepared through self-assembly processes in supramolecular chemistry [70,71,72,73] as well as artificial fabrication processes such as the self-assembled monolayer (SAM) method [74,75,76], Langmuir–Blodgett (LB) technique [77,78,79,80], and layer-by-layer (LbL) assembly [81,82,83]. These approaches have been thus far examined as separated and independent methodologies. In order to achieve more effective and versatile developments of materials exploration with nanoscale structural features, a unified methodology must be established as a post-nanotechnology concept. This task can be assigned as an emerging concept, nanoarchitectonics [84,85], which is an universal concept to fabricate functional materials from nanoscale building units (Figure 1) [86]. Like the historical proposal of nanotechnology by Richard Feynman [87,88], nanoarchitectonics was initially proposed by Masakazu Aono [89,90] in 2000 at the first International Symposium on Nanoarchitectonics Using Suprainteractions in Tsukuba, Japan.

Nanoarchitectonics is a conceptual methodology to combine nanotechnology with other research fields such as organic chemistry, supramolecular chemistry, materials chemistry, microfabrication technology, and bio-related science [91,92]. Functional material systems are prepared from nanoscale units such as atoms, molecules, and nanomaterials through combinations and selections of building units and processes including atom/molecular manipulation, chemical transformation, self-assembly/self-organization, field-controlled organization, material processing, and bio-related treatments [93]. Because this concept is general and applicable for a wide range of materials, the nanoarchitectonics concept has been used in various research fields such as material production [94,95,96], structural fabrication [97,98,99], catalysts [100,101,102], sensing [103,104,105], devices [106,107,108], environmental usage [109,110,111], energy-related applications [112,113,114], biochemical science [115,116,117], and biomedical applications [118,119,120]. Nanoarchitectonics strategies for materials creation from fundamental units of atoms and molecules could apply to any kind of material with any desirable function [121].

Nanoarchitectonics approaches have two distinct features. One of them is harmonized nature between contributing processes [122,123]. Unlike actions between objects at microscopic scales, the interaction between nanoscopic objects often includes uncertainties such as thermal fluctuations, statistical distributions, and quantum effects. Total effects are not always the same as a summation of individual actions. Therefore, materials productions have to be considered with the harmonization of contributing interactions rather than their simple summation. Another feature of the nanoarchitectonics approaches is advantageous features to construct asymmetric and/or hierarchical material systems [124]. Self-assembling processes are mostly driven through an energy–consume-less equilibrium. Unlike conventional self-assembling processes, the nanoarchitectonics approaches can include multiple steps where energy-consuming non-equilibrium processes are often involved. Stepwise processing and sequential treatment for materials fabrications result in the formation of materials with hierarchical structural motifs. It can be said that the nanoarchitectonics approaches are advantageous for the constraction of hierarchical materials structures.

Based on these backgrounds, fabrications of functional materials with hierarchical structural motifs from simple nano units of fullerenes (C_60_ and C_70_ molecules) are described in this review article. Because fullerenes can be regarded as simple and fundamental building blocks with mono-elemental and zero-dimensional natures, these demonstrations for hierarchical functional structures impress the high capability of the nanoarchitectonics approaches [125,126]. Especially, this review article exemplifies three classes of structures and functions of hierarchical fullerene materials: (i) hierarchically structured fullerene assembly for vapor sensor usage; (ii) fullerene assembly with microscopic recognition capability; (iii) fullerene microstructure with a mesoporous framework for advanced functions.

## 2. Hierarchically Structured Fullerene Assembly for Vapor Sensor Usage

**Figure 2:**
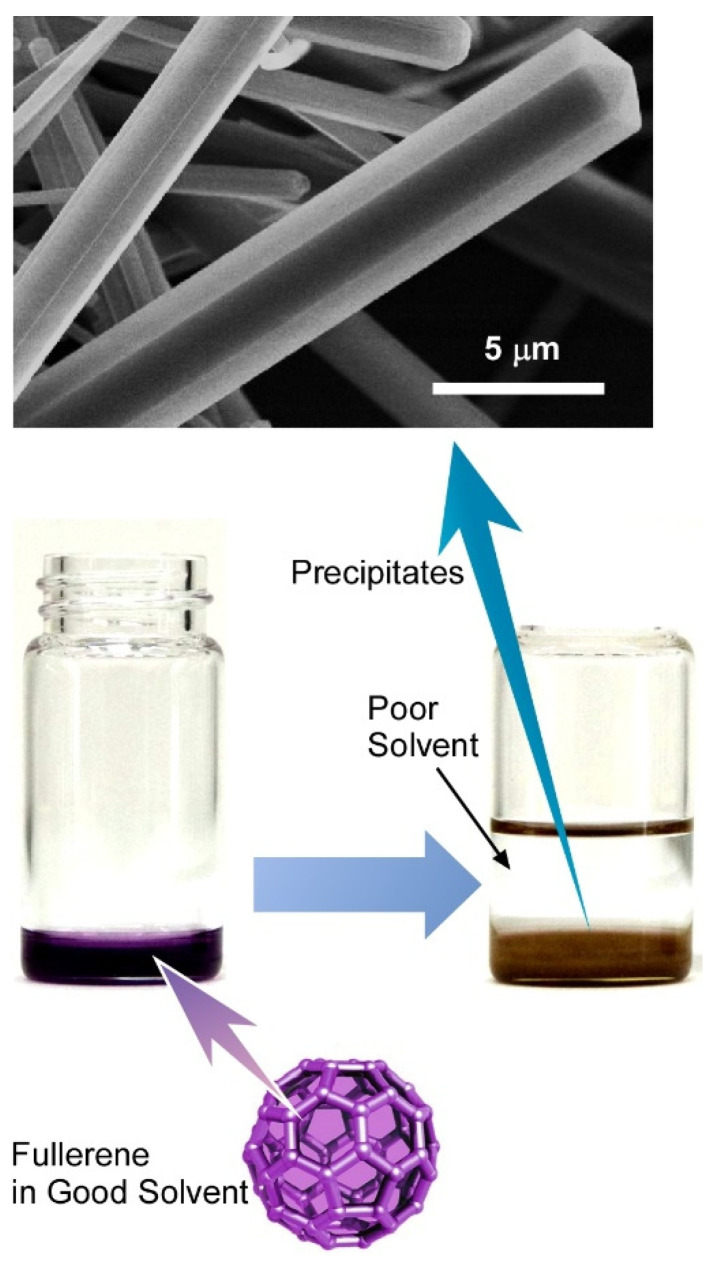
Liquid–liquid interfacial precipitation method for self-assembly of fullerene to form dimensionally-controlled nano or microstructures. An example to form a one-dimensional assembly is displayed.

Recently, hierarchical nanostructures have gained immense attention due to their multimodal versatility, such as porous architecture with high surface area, diverse functionality, synergistic interactions, multiple functionalities, and easy bottom-up synthesis method [127,128], which are in general difficult to achieve in cases of conventional nanomaterials. Owing to their versatility, hierarchical nanostructures have been used in areas of advanced applications, such as optoelectronics, energy harvesting, sensing, and photonics [129,130]. The selection of buildings blocks for hierarchical nanostructures is a challenging task and extremely important to obtain the desire materials for the specific application. In this regard, fullerenes (C_60_ or C_70_) have recently received significant interest due to their extended π-conjugation and applications in many advanced research fields such as biomedical, semiconductors, optics, electronics, and spintronics [131,132,133]. Additionally, fullerene readily undergoes self-assembly to form dimensionally-controlled nano or microstructures via the simple liquid–liquid interfacial precipitation method (Figure 2) [134,135]. Along with the liquid–liquid interfacial precipitation method, precise solvent engineering is also very important to control or transform the hierarchical fullerene structure. The fabricated hierarchical fullerene assemblies often exhibit high capabilities in vapor sensing.

### 2.1. Fullerene C_70_ Cube for Sensing Platform for Volatile Aromatic Solvent Vapor

**Figure 3:**
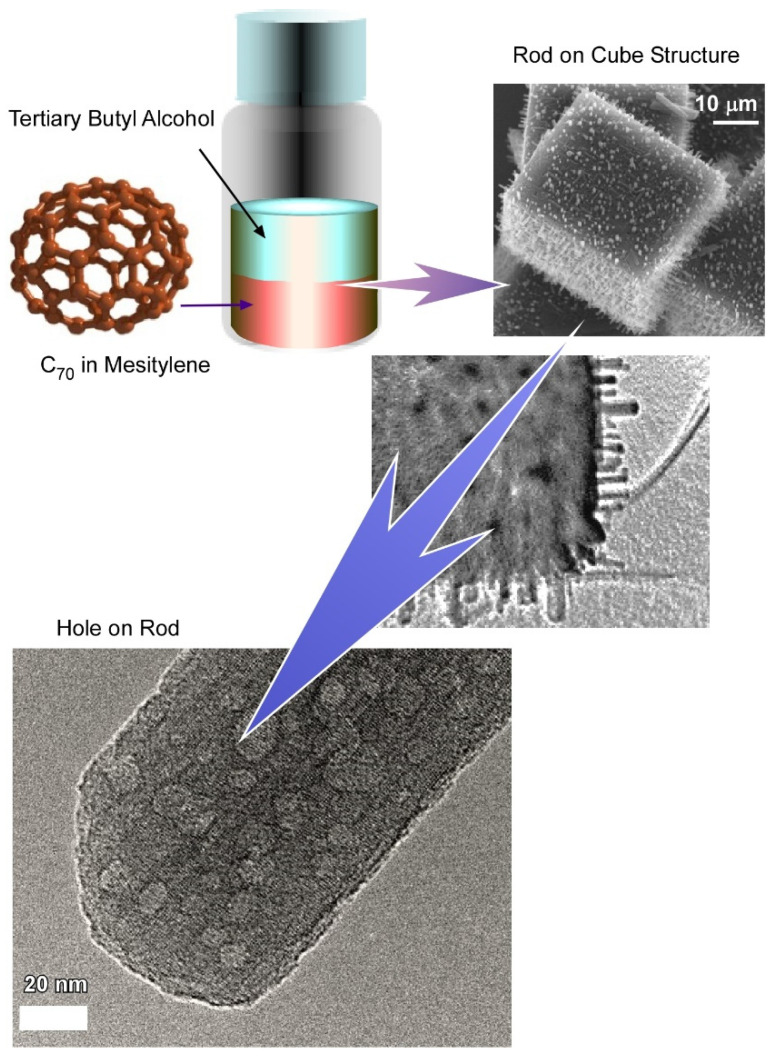
Preparation of hierarchically structured fullerene cubes and mesoporous fullerene C_70_ nanorods with crystalline pore walls. Reprinted with permission from Reference [136]. Copyright 2016 American Chemical Society.

Bairi et al. demonstrated the preparation of hierarchically structured fullerene cubes using C_70_ as building blocks through the liquid–liquid interfacial precipitation method (Figure 3) [136]. Structural analysis through scanning electron microscopy (SEM) and transmission electron microscopy (TEM) suggested that these cubes are composed of mesoporous fullerene C_70_ nanorods with crystalline pore walls, which make them an excellent candidate as a receptor layer for volatile solvent detection. Detailed study of the formation mechanism highlights the importance of precise solvent engineering to control such transformation of C_70_ fullerene cubes to hierarchically structured fullerene cubes. In this case, isopropyl alcohol was used as an additional solvent to complete this transformation process of C_70_ fullerene cubes to hierarchically structured fullerene cubes via handshaking, followed by incubation at 25 °C for 1 h. SEM observations confirmed that isopropyl alcohol triggers the structural changes of the cubes, resulting in the formation of fullerene C_70_ nanorods, which subsequently formed the cube surface. The high surface area and porous architecture of hierarchically structured fullerene cubes make them potential receptor materials for volatile organic compound sensing in combination with quartz crystal microbalance. These hierarchically structured fullerene cubes show excellent selectivity towards aromatic vapors over other organic volatile organic compounds due to the strong π–π interactions between host and guest. Sensitivity towards toluene is the highest among all the aromatic vapors. Additionally, sensitivity towards water vapor is very low, which is highly desirable for any kind of receptor material for gas sensing.

### 2.2. Dimension-Dependent Face-Selective Etching of Fullerene Assembly

**Figure 4:**
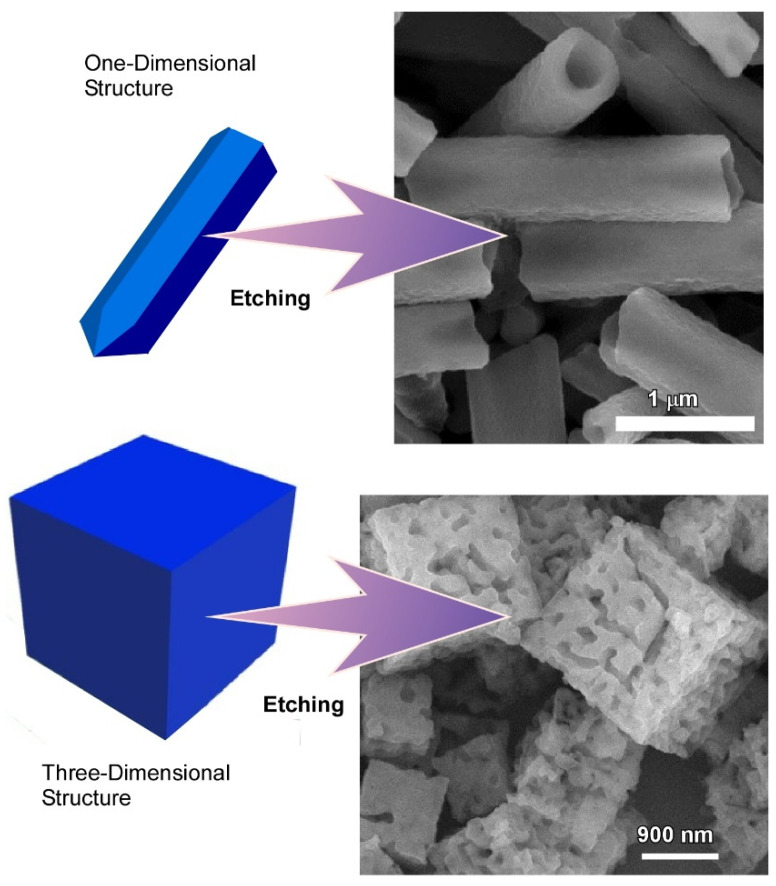
Formation of hollow and hierarchical fullerene nanostructures via face-selective etching of self-assembled fullerene crystals. Reprinted with permission from Reference [137]. Copyright 2020 Royal Society of Chemistry.

Controlled structural modification and surface functionalization of such hierarchical fullerene microstructure is highly desirable to widen their sensing ability towards nonaromatic vapors. Additionally, conversion of hydrophobic fullerene to their hydrophilic counterpart through surface functionalization opens the possibility to use them in several biological applications. Hsieh et al. demonstrated such modification through face-selective chemical etching of fullerene assemblies (Figure 4) [137]. Their results showed a simple and scalable strategy for the fabrication of hollow and hierarchical fullerene nanostructures via face-selective etching of the self-assembled fullerene crystals.

The fabrication of fullerene C_60_ nanorods, fullerene C_60_ nanosheets, and fullerene C_70_ cubes was demonstrated by using the modified liquid–liquid interfacial precipitation method called ultrasound-assisted liquid–liquid interfacial precipitation. SEM images revealed the formation of fullerene C_60_ nanorods, fullerene C_60_ nanosheets, and fullerene C_70_ cubes with a uniform size distribution. X-ray diffraction (XRD) confirmed the crystalline nature of the as synthesized fullerene assemblies. Chemical etching of these fullerene assemblies was performed in solution with ethylene diamine treatment for 10 min under ultra-sonication followed by incubation at 25 °C for 0 to 24 h depending on their shape. SEM observations revealed that ethylene diamine selectively etches at the ends of the one-dimensional nanorods, leading to the formation of hollow tubular structures, whereas for two-dimensional fullerene C_60_ nanosheets, ethylenediamine etches largely at their upper and lower surfaces and partially their edges. Similarly, ethylenediamine etches the faces of the three-dimensional cube, not the edges. However, there were no changes in the overall dimensions after etching indicated the perfect example of face-selective etching of the fullerene assemblies. Chemical analysis suggested that the etching of self-assembled fullerene assemblies is due to the amination reaction between the primary amine, ethylenediamine, and π-electron-rich fullerene. Additionally, the chemical etching of post-assembled fullerene crystals is highly dependent on the co-solvent as well as different crystal forms. This effect can be attributed to the difference in solubility of fullerene in different co-solvents. On the other hand, face-selectivity can be explained by the different reactivity of the different faces of the as-prepared fullerene assemblies.

This kind of chemical etching leads to changes of fullerene assembly surfaces from hydrophilic to hydrophobic. Additionally, porosity is also improved by such chemical etching, which leads to better sensing performance. Selectivity of these hydrophilic fullerene assemblies towards hydrophilic acidic volatile components is higher than that of aromatic volatile organic compounds due to the favorable interaction with the amine group, although, selectivity towards other hydrophilic solvents vapor like alcohol is very low. Additionally, such water-dispersible fullerene assemblies are highly important for their use in biological fields.

### 2.3. Bitter Melon Shaped Nanoporous Fullerene C_60_ Assembly

**Figure 5:**
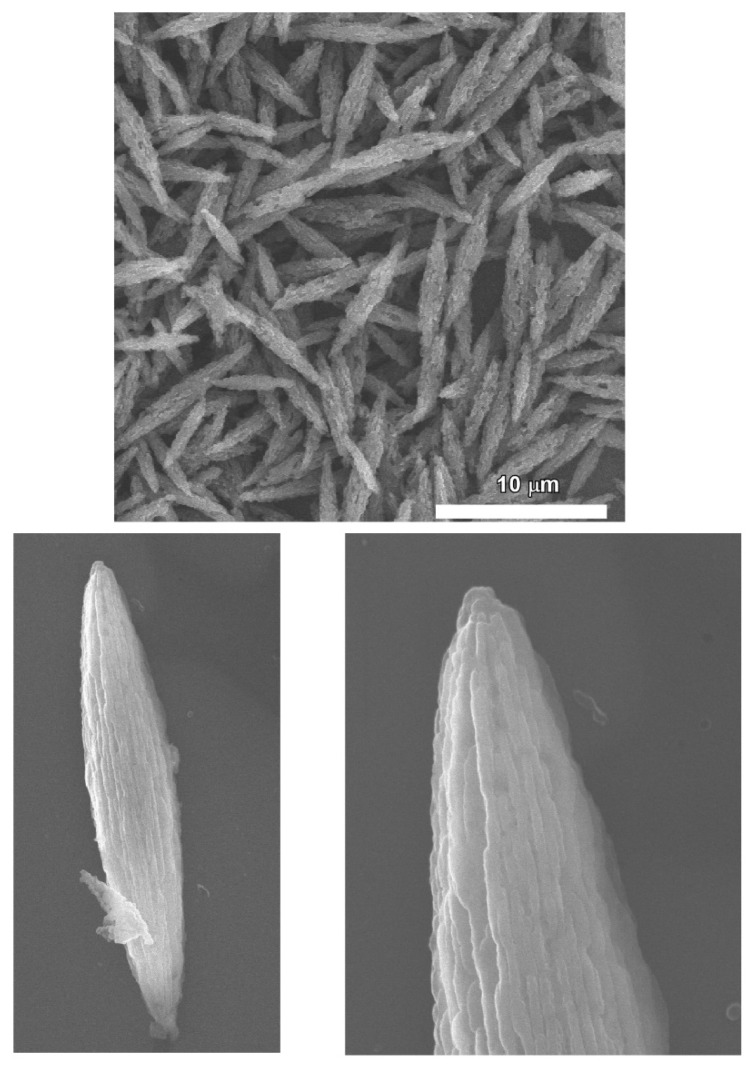
“Bitter melon” shaped nanoporous C_60_ assemblies prepared through the liquid–liquid interfacial precipitation method at room temperature [138].

As a continuation of the previous discussion, not only the surface functionality but also the shape of the fullerene hierarchical nanostructures has great importance to improve their potential application in a different field. In this regard, the combination of good solvent and poor solvent during the self-assembly of pristine fullerene via the liquid–liquid interfacial precipitation method is the determining factor to control the hierarchical structure. Furuuchi et al. demonstrated the assembly of C_60_ into exceptional morphology called “bitter melon” shaped nanoporous C_60_ assemblies by tuning the liquid–liquid interfacial precipitation method at room temperature (25 °C) (Figure 5) [138]. In this case, isopropyl alcohol was used as a poor solvent, and C_60_ solution in dodecylbenzene as a good solvent to form a clear liquid–liquid interface. Ultrasonication and vortex mixing were applied to modify the conventional liquid–liquid interfacial precipitation method before 24 h incubation at 25 °C. XRD and high-resolution transmission electron microscopy (HRTEM) analysis confirmed the crystalline nature of the self-assembled fullerene structure. Powder XRD patterns of the as synthesized “bitter melon” shaped C_60_ assemblies showed mixed crystal phases, including *fcc* and *hcp* phases. TEM analysis confirmed the nanoporous nature of the fullerene structure. Surface textural properties and nanoporous architectures of bitter melon shaped fullerene C_60_ assemblies make them excellent receptor materials of quartz crystal microbalance for sensing toxic volatile organic compounds. Here also, selectivity towards aromatic solvent vapors is excellent over nonaromatic compounds due to the favorable π–π interaction. Among aromatic vapor, selectivity towards aniline is the highest.

Moreover, micro or mesoporous self-assembled hierarchical fullerene nanomaterials are a class of materials that display excellent efficiency as volatile organic compound sensors, with selectively for aromatic vapors due to their extended π-conjugated structure and favorable π–π interaction with the aromatic vapors. Additionally, functional modification from hydrophobic to hydrophilic while maintaining the morphology improves their selectivity from aromatic to nonaromatic solvent vapor.

## 3. Fullerene Assembly with Microscopic Recognition Capability

The concept of the nanoporous hierarchical structure has been studied extensively and is now well established. However, the manipulation of micron-sized hollow objects with self-assembled hierarchical structures remains very challenging. Such kinds of microscale hollow objects and manipulation of such hollow structures are highly important for advanced applications, which include loading, transportation, and release of nano/micron-sized objects, especially cells, bacteria, biomolecules, and functional nanoparticles [139,140,141]. Therefore, researchers are giving extensive effort to fabricate such micron-sized hollow hierarchical objects, and there are only a few successful examples. In the following examples, fullerene C_70_ offers such manipulable micron-sized hollow structures via the controlled liquid–liquid interfacial precipitation method.

### 3.1. Hole-in-Cube Fullerene Assembly with Microscopic Recognition Capability

**Figure 6:**
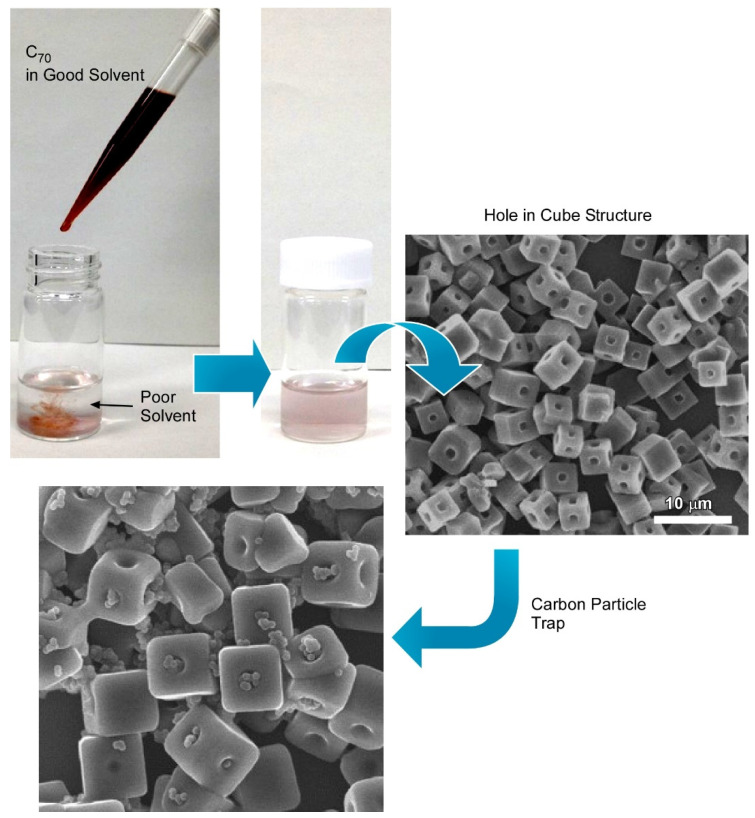
Fabrication of C_70_ cube with open hole on each face of the cube (hole-in-cube structure) through controlled self-assembly at the liquid–liquid interface and trapping capability for carbon particles. Reprinted with permission from Reference [142]. Copyright 2017 American Chemical Society.

Bairi et al. demonstrated the fabrication of a C_70_ cube with an open hole on each face of the cube through controlled self-assembly at the liquid–liquid interface (Figure 6) [142]. Additionally, it was established that the process to close and open the holes can be done purposefully. Fullerene C_70_ cubes with open or closed holes were produced by the dynamic liquid–liquid interfacial precipitation method at 25 °C using mesitylene as a good solvent and tertiary butyl alcohol as a poor solvent followed by 24 h incubation under 25 °C. The formation mechanism of such an open hole cube is different from the previously discussed solvent or chemical etching mechanisms. Detailed structural analysis via cross-section SEM confirmed that the holes are not hollow through. Additionally, TEM images of fullerene assemblies formed just after the mixing of C_70_-mesitylene with tertiary butyl alcohol confirmed the formation of a smaller cube with no holes. It was suggested from this observation that the open hole formation is not driven by the solvent etching mechanism, but rather the growth of open hole cubes involves a two-step process including a solid core formation at the first step followed by slow growth to the final open hole cube formation. In the second step, concentration depletion and the different reactivity of the corner, as well as the edges, play important roles to form such a uniform open hole cube. This observation is in line with the formation mechanism of the closed hole cube. When the mesitylene/tertiary butyl alcohol ratio is fixed at 1:2, the C_70_ concentration is below the critical concentration level to form the new core. Therefore, C_70_ molecules tend to grow over each face of the open hole cube to form the closed hole cube.

These open hole cubes show excellent microscopic recognition properties towards the micron size particles. Open hole cubes specifically recognize the graphitic carbon particles over resorcinol–formaldehyde polymeric resin particles with similar dimensions. SEM observations show that the open holes of the cube were mostly occupied by the graphitic particles, whereas polymeric resin particles were not recognized by these open hole cubes. This phenomenon can be attributed to the favorable π–π interaction of the graphitic carbon particles with the fullerene cube. Moreover, controlled synthesis of such functional materials with precise manipulation of open or closed hollow structures is highly important for many advanced applications, such as the controlled release of drugs, protection of biologically active species, and removal of pollutants from air or water.

### 3.2. Fullerene Microhorns with Microscopic Recognition Properties

We discussed in the previous section that the manipulation of the hollow structure with specific morphology is highly important for advanced nanotechnology. In addition to this, the structural transformation from one morphology to another is also extremely important. It is very important to find out an effective and versatile strategy to manipulate the morphology of such self-assembled nanostructures. In this regard, solvent engineering is showing extreme potential, which involves the use of different types of solvents or a mixture of solvents with different ratios to control the self-assembly conditions to achieve desired materials.

**Figure 7:**
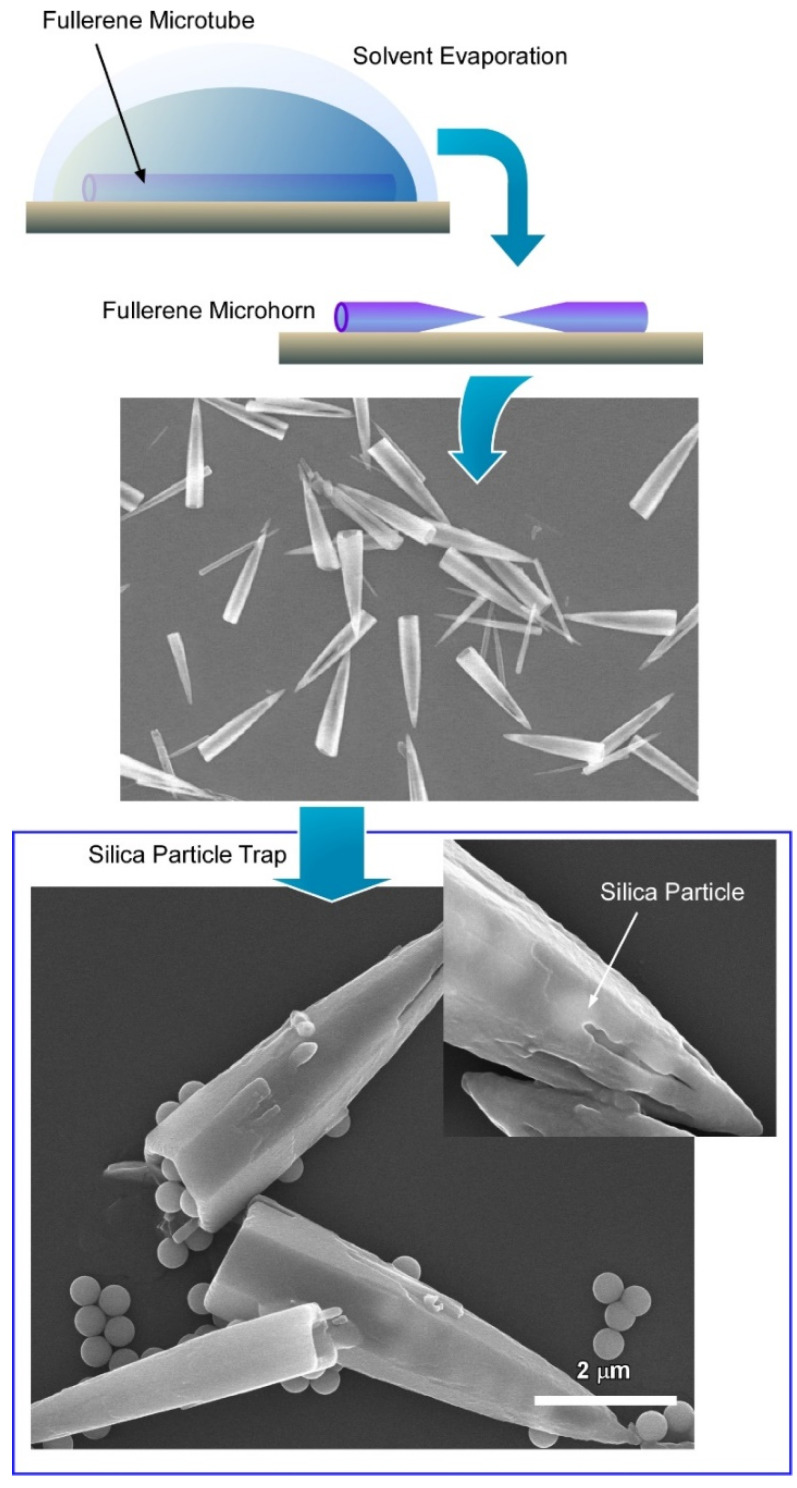
Formation of conical-shaped fullerene microhorn from fullerene microtube and trapping behavior of silica particles. Reprinted with permission from Reference [143]. Copyright 2019 American Chemical Society.

Recently, Tang et al. demonstrated the fabrication of fullerene microstructures with a hollow framework from a mixture of C_60_ and C_70_ based on the dynamic liquid–liquid interfacial precipitation method (Figure 7) [143]. Additionally, considering the different crystalline phases and solubility of C_60_ and C_70_ and precise solvent engineering, they can transform the microstructure into unique conical-shaped fullerene microhorns. A C_60_ and C_70_ fullerene mixture with a 4:1 volume ratio was used in mesitylene as a good solvent. A mixed fullerene microtube was fabricated by the dynamic liquid–liquid interfacial precipitation method using tertiary butyl alcohol as a poor solvent. The overall reaction was very fast and completed in a few seconds after the addition of the fullerene fixture into the poor solvent. Details analysis suggests that the mixing ratio of fullerene C_60_ and C_70_ plays an important role to control the formation of the microtube. Interestingly, the microtube formation of fullerene C_70_ is not possible due to their crystal packing. However, with the help of fullerene C_60_, fullerene C_70_ was forced to form the microtube through hexagonal closed packing. Fullerene microtube-to-microhorn transformation was accomplished by precise solvent engineering. Then, a mesitylene/tertiary butyl alcohol (volume ratio of 1:3) solvent mixture was used to wash the microtube to form the perfectly homogeneous microhorn. Interestingly, there was no change in crystallinity of the microhorn, which suggests that the process of such morphological transformation is fully dominated by the physical changes. Additionally, such solvent engineering helps us to generate a nanoporous architecture inside the microhorn.

Real-time optical microscope imaging suggests that the formation of microhorns directs through the solid core dissolution of the fullerene microtube. This can be explained by the difference in solubility in good and poor solvents. Solubility of fullerene C_70_ is lower in alcohol than that of fullerene C_60_. Therefore, fullerene C_70_ molecules tend to form tiny aggregates when the mixture of C_60_ and C_70_ starts to form the crystal, and that core forms the center solid part of the microtube. Therefore, there is a higher concentration of C_70_ at the central part of the microtube. When the mesitylene–tertiary butyl alcohol solvent mixture is added to the microtube, C_70_ molecules tend to dissolve faster in mesitylene due to their higher solubility over C_60_. In this way, these fullerene microtubes transfer to their secondary morphology microhorn.

These hollow microhorns also show excellent microscopic recognition properties to the silica particles over polystyrene particles. However, the recognition property towards fullerene C_70_ nanoparticles is not so satisfactory, which suggests that this phenomenon is not directed by typical π–π interactions between the microhorn and the C_70_ particles. Zeta potential charge analysis suggests that the microscopic recognition phenomenon is governed by the electrostatic surface charge of the microhorn. Moreover, such morphological transformation of fullerene assemblies through solvent engineering delivers important perceptions into the field of supramolecular self-assembly. Additionally, specific microscopic recognition by such hollow structures has immense potential for utilization in different fields, as stated earlier.

## 4. Fullerene Microstructure with Mesoporous Framework for Advanced Function

Not only the microscopic hollow structure but also the mesoporous structural materials with crystalline frameworks also have great importance in many advanced fields. Additionally, such post-synthesis modification of self-assembled materials to their mesoporous architecture critically influences their overall properties including optical, electrical, charge storage, etc. Thus, mesoporous materials with crystalline frameworks and tunable pore sizes might have substantial technological advantages. Therefore, a proper synthesis methodology and suitable building blocks for novel crystalline mesoporous materials remain a high point of interest due to their significant potential in many advanced fields. Here, fullerene also plays an important role to form such a crystalline framework via self-assembly, and transformation to the mesoporous framework can be achieved by a simple post-synthesis treatment, which leads to changes in its optical, charge storage mechanism.

### 4.1. Mesoporous Fullerene C_70_ Cube with Enhanced Photoluminescence Property

Bairi et al. have recently demonstrated one such novel piece of work, which includes the formation of mesoporous fullerene C_70_ cubes with highly crystalline frameworks with excellent photoluminescence properties [144]. Crystalline fullerene cubes with sharp edges were fabricated by the ultrasound liquid–liquid interfacial precipitation method from tertiary butyl alcohol as a poor solvent and a solution of C_70_ in mesitylene as a good solvent at 25 °C. A good solvent to poor solvent ratio is one of the important parameters to control the desired morphology, and here in this case it was 1:5. After the preparation of the fullerene cube, the mother liquor was stirred at 300 rpm for 72 h at 75 °C and then drop-casted on a silicon wafer and dried at 80 °C to prepared the mesoporous crystalline fullerene cube. An adsorption isotherm confirmed the formation of mesopore inside the fullerene cube, as the surface area of the mesoporous structure was higher than that of the fullerene cube. Crystal structure analysis via XRD confirmed that the as-synthesized fullerene cube contained the simple cube packing, whereas mesoporous has a mixed crystal phase with a simple cube and hexagonal closed pack. This phenomenon can be attributed to the entrapped solvent molecules, which change the crystal packing of C_70_ and reducing the crystal symmetry. Photoluminescence properties of mesoporous fullerene cubes are improved by such crystallographic modification, which can be confirmed from the comparison of photoluminescence spectra of pristine C_70_, fullerene cubes, and mesoporous fullerene cubes. It is worth noticing that for π-conjugated molecules, photoluminescence intensity is quenched in the solid-state. However, the photoluminescence intensity of mesoporous fullerene cubes is higher in the solid-state, which indicates the importance of such structural and crystalline framework modification.

### 4.2. Mesoporous Carbon Cubes for Supercapacitors

We have discussed earlier that the incorporation of mesopores creates substantial changes in material properties and makes them suitable for many advanced applications. One such application of the mesoporous materials is in electrochemical charge storage. During the last three decades, researchers have given enormous efforts to finding suitable mesoporous materials for charge storage applications [145,146,147]. Carbon-based materials with mesoporous architectures are supposed to be the best materials for such charge storage properties [148,149,150]. However, unwanted surface functionality and poor electrochemical conductivity of such carbon materials have to be addressed to improve their performance. Fullerene C_60_ or C_70_ is a π-conjugated molecule and readily self-assembles to form a homogeneous shape-controlled microstructure. Recently, researchers have focused on these dimensionally-controlled fullerene assemblies as an exceptional source of π-electron-rich carbon materials. Such extended conjugated π-systems with high surface areas and mesoporous frameworks will be advantageous for energy storage applications such as supercapacitors, battery, etc. [151,152,153].

Bairi et al. demonstrated the direct transformation of porous crystalline fullerene C_70_ cubes into mesoporous carbon cubes that possess a very high specific surface area [154]. Synthesis of a porous crystalline C_70_ cube was done by the liquid–liquid interfacial precipitation method followed by mild heat treatment at 70 °C. XRD and TEM analysis confirmed the crystalline framework of the C_70_ cube. Conversion of porous crystalline fullerene C_70_ cubes to high surface area mesoporous carbon cubes was performed by high-temperature heat treatment (900 °C) under a continuous flow of N_2_ gas in a tube furnace. A nitrogen adsorption isotherm confirmed the formation of a mesoporous architecture with a narrow pore size distribution. The Brunauer–Emmett–Teller surface area of the mesoporous carbon cube was very high at ca. 642.6 m^2^ g^−1^, which is almost 14 times higher than that of the porous crystalline fullerene cube (ca. 47.7 m^2^ g^−1^). Therefore, high-temperature heat treatment is an essential step for such a large improvement of specific surface area. Pore size analysis by Barrett–Joyner–Halenda and the non-local density functional theory method confirmed the presence of both micropores and mesopores. The average pore size of the obtained carbon cube was 3.44 nm with a high pore volume of 0.367 cm^3^ g^−1^. XRD and Raman analysis of the obtained carbon cube confirmed the graphitic nature of carbon.

This newly synthesized mesoporous carbon cube with a high surface area showed excellent electrochemical charge storage properties. Electrochemical measurements via cyclic voltammetry and charge–discharge measurements revealed that this microstructure carbon cube showed excellent specific capacitance of ca. 286 F g^−1^ at a scan rate of 5 mV s^−1^ and 205 F g^−1^ at a current density of 1 A g^−1^. These values are highly comparable with other nanocarbon materials, such as graphene and carbon nanotubes. The rate capability of this material is excellent, with a very high retention of specific capacitance of 56.0% at a very high current density of 20 A g^−1^. Additionally, the cyclic stability of this material is also very high, with almost no loss of specific capacitance even after 10,000 charge–discharge cycles.

### 4.3. Quasi Two-Dimensional Mesoporous Carbon Microbelts for Supercapacitors

Electrochemical charge storage is highly dependent on the effective surface area accessible to the electrolyte ions. Therefore, it is very important to change the morphology of the fullerene-derived carbon to obtain maximum charge storage capacity. Here, fullerenes offer a great opportunity, as they can form almost any kind of morphology starting from one-dimensional rod to two-dimensional sheet to three-dimensional cube through the supramolecular assembly. Studies on charge storage mechanisms suggest that the sheet-like structure shows great performance.

**Figure 8:**
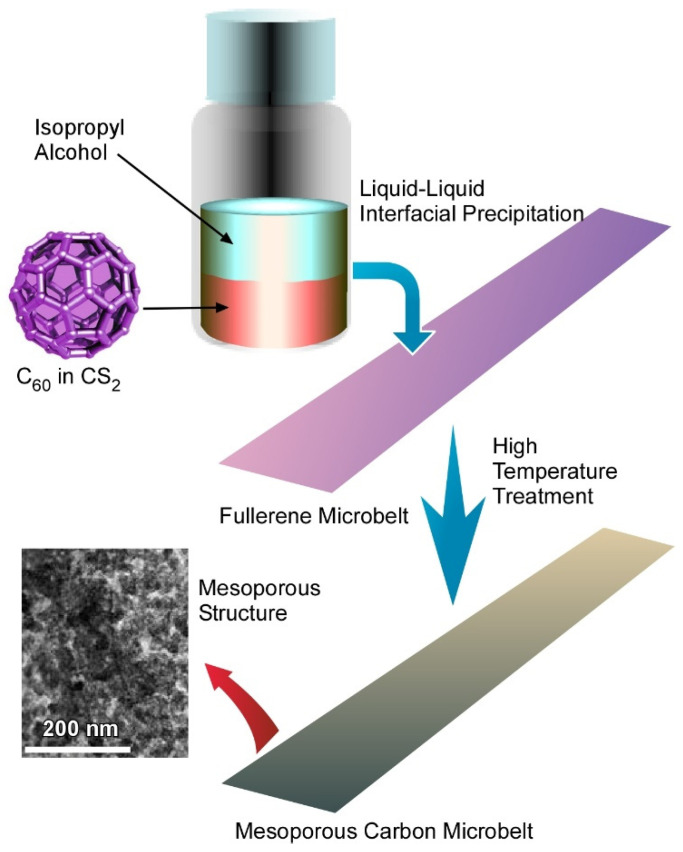
Fabrication of two-dimensional fullerene microbelts through transformation of fullerene assembly to mesoporous carbon with retention of their original sheet-like structure. Reprinted with permission from Reference [155]. Copyright 2017 American Chemical Society.

Recently, Tang, et al. reported a novel method for fabrication of two-dimensional fullerene microbelts, which can be transferred to mesoporous carbon with the retention of their original sheet-like structure (Figure 8) [155]. Fullerene microbelts were made using the conventional liquid–liquid interfacial precipitation method taking CS_2_ as a good solvent and isopropyl alcohol as a poor solvent followed by 24 h incubation at 25 °C to complete the full growth of the fullerene microbelts. Powder XRD analysis of the fullerene microbelts confirmed the mixed *fcc* and monoclinic crystal phase, which is different from the *fcc* crystal phase of the pristine C_60_ powder. Mesoporous carbon microbelts were prepared by high-temperature heat treatment at the two different temperatures of 900 °C and 2000 °C. A nitrogen adsorption isotherm confirmed the formation of the porous architecture of the fullerene microbelt-derived carbon. Brunauer–Emmett–Teller surface areas of mesoporous carbon microbelts derived at 900 °C and 2000 °C were 980 m^2^ g^−1^ and 297 m^2^ g^−1^, respectively. The surface area of the fullerene microbelt was much lower compared to that of the fullerene microbelt-derived carbon materials, which confirmed the importance of high-temperature heat treatment. Raman and XRD analysis confirmed the graphitic nature of the derived mesoporous carbon belt. TEM and HRTEM analysis of the derived mesoporous carbon confirmed the amorphous nature of the carbon.

These mesoporous microbelts were used as active electrode materials for supercapacitive charge storage applications. Cyclic voltammetry curves of the mesoporous carbon microbelts showed quasi-rectangular shapes even at a very high scan rates, which indicated the typical feature of electrical double-layer capacitors. The specific capacitance of mesoporous carbon microbelts obtained from cyclic voltammetry curves was 360 F g^−1^ at 5 mV s^−1^, which was very high compared to the fullerene microbelts (38.4 F g^−1^at 5 mV s^−1^). This improvement in specific capacitance can be attributed to the high surface area of the mesoporous carbon microbelts. Chronopotentiometry (charge–discharge) curves of the mesoporous carbon microbelts showed a triangular nature, which is also the signature of the electrical double-layer capacitors. The specific capacitance obtained from the charge–discharge curves for mesoporous carbon microbelts prepared at 900 °C was 290 F g^−1^ at a current density of 1 A g^−1^. Additionally, these derived carbon materials showed excellent rate capability with retention of 48.8% specific capacitance even at a very high current density of 10 A g^−1^. A cyclic stability test of the mesoporous carbon microbelts prepared at 900 °C was performed over 10,000 charge–discharge cycles at 10 A g^−1^, which showed positive cyclic stability. This phenomenon can be recognized as additional activation of intercalation or deintercalation of electrolyte ions during the charging/discharging cycles.

## 5. Summary and Perspectives

This review article summarized several examples focused on syntheses and function explorations of fullerene assemblies with hierarchical and asymmetric structural features. Basic structures of these fullerene assemblies are fabricated first through very simple methods, mostly the liquid–liquid interfacial precipitation method, and then post-treatment often results in conversion of rather simple symmetric structures to complicated hierarchical structural motifs. Process-integrated features in nanoarchitectonics approaches are advantageous for the preparation of hierarchical functional structures. Even though very simple fullerenes with mono-elemental (carbon) zero-dimensional structures are used as building units, various hierarchical structures such as cubes with nanorods, hole-in-cube assemblies, face-selectively etched assemblies, and microstructures with mesoporous frameworks are fabricated upon easy fabrication protocols. Accordingly, the fabricated fullerene assemblies are used in various applications including volatile organic compound sensing, microparticle catching, supercapacitors, and photoluminescence systems.

It must be noted that these rich varieties of structures and functions are obtained through nanoarchitectonics processes of simple building units. Expansion of these methodologies to a wide range of materials will create further huge possibilities in the fabrication of functional materials with hierarchical structural motifs. Various structural units with specific interactions such as host–guest systems [156,157,158,159] and biomolecules (and their mimics) [160,161,162] would have high potentials as active building blocks. In addition, the other material-based building units [163,164,165] and their hybrids/composites [166,167,168] would open further possibilities in nanoarchitectonics materials predictions. This wide range of possibilities could be logically handled by emerging approaches such as machine learning [169,170,171].

This review article mainly discusses recent progress on hierarchical fullerene nanoarchitectonics, mainly from our own results that would have some progress from the first step fullerene assemblies [172]. Not limited to our results, some examples on fullerene assemblies with certain complexities have been reported from some groups [173,174,175]. Various factors including molecular designs and assembling conditions would effectively work on the construction of desired structures, which is not fully clear yet. Accumulation of research facts would lead to total understanding of hierarchical fullerene nanoarchitectonics in the near future. From basic science to practical applications such as biomedical usages, various outputs can be highly expected.
